# Illicit Cigarette Consumption and Government Revenue Loss in Vietnam: Evidence from a Primary Data Approach

**DOI:** 10.3390/ijerph16111960

**Published:** 2019-06-02

**Authors:** Minh T. Nguyen, Son The Dao, Nga Que Nguyen, Mike Bowling, Hana Ross, Anthony D. So

**Affiliations:** 1 Department of Economics, Ball State University, Muncie, IN 47306, USA; 2 Department of Economics, Thuongmai University, Hanoi 10000, Vietnam; daotheson@tmu.edu.vn; 3 Vietnam Academy of Social Sciences, Hanoi 10000, Vietnam; nguyenquenga@gmail.com; 4 Department of Health Behavior, University of North Carolina-Chapel Hill, Chapel Hill, NC 27599, USA; jbowling@email.unc.edu; 5 School of Economics, University of Cape Town, Rondebosch 7700, South Africa; hana.ross@uct.ac.za; 6 Innovation+Design Enabling Access (IDEA) Initiative, Department of International Health, Johns Hopkins Bloomberg School of Public Health, Baltimore, MD 21205, USA; anthony.so@jhu.edu

**Keywords:** Illicit trade, tobacco, tax avoidance, cigarette smuggling, cigarette price, primary data, pack examination, Vietnam

## Abstract

This article provides the first comprehensive picture and independent estimates of both illicit cigarette consumption and the resulting government tax revenue loss in Vietnam using data from a representative survey of cigarette smokers in 12 Vietnamese provinces. The survey consisted of face-to-face interviews and on-site cigarette pack examinations. We find that more than 720 million illicit cigarette packs, or 20.7% of total cigarette consumption, circulated in Vietnam in 2012. Consequently, government tax revenue loss due to illicit trade ranged from US $223 to 295 million. Our estimates also indicate that (1) the most popular illicit brands were Jet and Hero, both were sold at higher prices than the average legal brand; (2) the average price of illicit cigarettes was 51% higher than the average price of legal cigarettes; and (3) majority of illicit cigarettes were sold at convenience stores, which were registered and licensed businesses. Our findings suggest that prices are not a driver of illicit cigarette consumption in Vietnam, and this illicit trade is at least partially a consequence of weak market control enforcement.

## 1. Introduction

The illicit trade in tobacco products is of interest to both governments and public health circles. Governments are concerned because tax revenues suffer from the avoidance and evasion of cigarette taxes. Illicit activities are known to undermine the government’s authority, and money from illicit trade is often reinvested in other criminal activities [1]. Public health specialists are concerned because by increasing the accessibility and affordability of tobacco products, illicit cigarette sales likely undermine the effectiveness of price and tax measures that aim to reduce consumption. Illicit sales also weaken other public policies, such as regulations on health warnings, packaging, and ingredients, since illicit cigarettes are not likely to follow any of those regulations [2,3]. On the other hand, the tobacco industry has consistently claimed that tax increases, graphic health warnings, restrictions on packaging, and regulations of ingredients are all drivers of the illicit cigarette trade [4]. The use of black-market cigarettes as leverage against the adoption of tobacco control policies [5] highlights the importance of understanding the scope and characteristics of the illicit trade in cigarettes. Unfortunately, the literature on the magnitude and characteristics of illicit cigarettes is thin [6,7,8], though the lack of research is understandable. The illegal nature of black-market trade activity makes it hard to track and monitor. Even when researchers obtain data on illicit cigarettes, there are challenges to estimating the scope of illicit products because the data are often not representative [6]. 

This article quantifies the magnitude of smuggled cigarettes in Vietnam, provides estimates of the amount of government cigarette tax revenue lost to the black market, and characterizes illicit cigarettes in terms of their prices, sources of origin, and points of sale. We surmounted the challenges usually facing researchers by combining several credible and recommended methods of measuring illicitly traded products. We conducted a representative survey of illegal cigarette consumption, the Vietnam Illicit Trade Assessment (VITA), in 12 provinces, in which the identification of illicit packs was based on clear and objective criteria. The problems of validity and underreporting that commonly afflict self-reported data were eliminated through two rounds of examination of the cigarette packs most recently purchased by smokers. 

The article makes several contributions to the literature. This paper is the first to provide a comprehensive picture and independent estimate of illicit cigarette consumption in Vietnam. Secondly, as the first independent estimate of the volume of illicitly traded cigarettes in Vietnam, the study serves as a tool to examine whether the tobacco industry has overstated the scope of illicit cigarettes in the country. Previous estimates used for policy development were generated by a group of domestic, state-owned cigarette manufacturers—the Vietnam Tobacco Association (VTA)—which has a vested interest in exaggerating the size of the black market. Third, it provides indirect evidence that taxation has not been a driver of cigarette smuggling in Vietnam. And finally, because our survey was conducted before the establishment of Health Promotion and Tobacco Control Fund and before the regulation of graphic health warnings came into effect in Vietnam, it serves as a baseline benchmark for illicit cigarette use, which could be used together with any later surveys in order to evaluate how the volume and characteristics of the illicit trade in cigarettes have changed over time. 

## 2. Background on Cigarette Taxation and Smuggling

In 2012, the year we conducted our survey, cigarette products in Vietnam were subject to three types of taxes: excise tax, value-added tax (VAT), and an import tax that applies to foreign-made products only. The excise tax was first introduced in 1993 and has gone through several reforms. Prior to 2006, Vietnam applied a complex excise tax system, in which different tax rates applied to cigarettes with different characteristics (see Appendix A, Table A1 for details). Since 2006, Vietnam has imposed a single ad valorem (value-based) excise tax rate on the ex-factory price on all cigarettes. The tax rate follows a roadmap set by the Vietnam Ministry of Finance and has slowly increased over time: 55% in 2006, 65% in 2008, 70% in 2016, and 75% in 2019. Although the statutory excise tax rate was 65% of the ex-factory price in 2012, the actual rate on the retail price comes to only approximately 32%. 

Other types of taxes on tobacco in Vietnam have not changed over the last several decades. A VAT of 10% of the retail price was introduced in 1993 [9]. Combined together, the effective tax rate applied on a typical pack of domestic cigarettes in Vietnam in 2012 amounted to 41% of the retail price, well below both the global average share of tax (52%) and the WHO’s recommended rate (excise tax of 75% of the retail price) [10]. 

Cigarette smuggling has been a concern of the Vietnamese government for many years. Prior to 2006, sales of foreign-made cigarettes were banned in the country. Cigarette smuggling circumvented this obstacle and became a source of foreign-made cigarettes for domestic consumers. In 2006, Vietnam joined the World Trade Organization and consequently opened its market to foreign cigarette manufacturers. However, the import of foreign-manufactured cigarettes has faced quota restrictions and is subject to an import tax rate of 135% of the CIF (cost, insurance, and freight) price prior to the excise and VAT taxes. 

The only estimates of the quantity of cigarettes smuggled into Vietnam have been coming from the Vietnam Tobacco Association (VTA), a group of domestic tobacco manufacturers. This group also funds governmental enforcement efforts against smuggling. According to the VTA’s report, smuggled cigarettes accounted for approximately 22% of the total cigarette market in 2012. The report contended that the black market was driven partially by increased excise taxes, and that the illicit trade would even grow larger if regulations on graphic health warnings were applied [11]. There has been no independent research on either the magnitude or the distribution channels of smuggled cigarettes in Vietnam. The government’s enforcement efforts against the smuggling of tobacco, including the Border Army, have normally monitored and seized illicitly traded cigarettes along the border between Vietnam and other countries [10].

## 3. Methodology

### 3.1. Vietnam Illicit Trade Assessment 2012

#### 3.1.1. Questionnaire

Our survey, the Vietnam Illicit Trade Assessment (VITA) 2012 was the first survey designed specifically to obtain information on illicit cigarettes and to provide estimates on the share of illicit cigarette consumption in Vietnam. 

In order to identify illicit packs, VITA 2012 included an on-site cigarette pack observational section. Government officials in charge of market control at the Ministry of Industry and Trade were consulted during the development of this section. The Vietnamese law required legal cigarette packs, whether domestic or foreign-made, to include tax stamps (a sales tax stamp for domestic packs and an import tax stamp for foreign-made packs), health warnings in Vietnamese, and information on the location of the manufacturer in Vietnamese. The absence of any of these details or fabricated tax stamps was considered to indicate that the pack was illicit. Trained interviewers inspected the cigarette pack most recently purchased by interviewees and filled in this observational form to capture important pack characteristics that were used later to verify whether the pack was illicit. These packs were collected at the end of the interview for later verification by the core team using a data collection instrument covering the same items as in the field interviewer’s questionnaire. In addition, tax stamps were authenticated using electronic readers. The data from both instruments were independently entered, and wherever there was a discrepancy, the cigarette pack collected from the household surveys was re-examined to verify that the correct information was entered.

Questions such as “on average, how many cigarettes do you smoke per week”, “how much was the price of the cigarette pack in your last purchase”, and “where did you buy your cigarettes in your last purchase” were used to calculate the annual consumption of cigarettes and prices per pack, and to determine their distribution channels. A copy of the questionnaire is available from the authors upon request.

#### 3.1.2. Sampling and Data Collection

VITA 2012 is a representative survey of cigarette smokers in 12 provinces of Vietnam. We selected the 12 provinces to capture a range of geographic and socioeconomic conditions of the country. Vietnam’s 63 provinces were grouped into three geographic and socioeconomic regions, whose characteristics were considered to differ substantially from each other, namely: the North, the South, and the Central regions of the country. Four provinces from each region were chosen randomly. Each selected province included urban and rural areas. The 12 selected provinces—Hanoi, Bac Giang, Phu Tho, Ninh Binh, Quang Binh, Da Nang, Lam Dong, Dak Lak, Ho Chi Minh City, Long An, Binh Phuoc, and Dong Thap—constitute 32% of the total population of Vietnam. While not a random sample selected nationally, this stratified sampling design ensured that the sample included regional differences across the country. We discuss the representativeness of our survey when we present our estimates side-by-side with estimates from the Global Adult Tobacco Survey (GATS) 2010, a nationally representative survey, in Section 4.2. 

The sampling strategy within selected provinces was similar to that used in the GATS [12], although the populations targeted in the two designs differed. GATS targeted all non-institutionalized adults aged 15 and over, whereas our survey used a random sample of smoking adults 18 years old and above. A two-stage sampling design was followed in which geographical clusters of households in the 12 provinces were selected in the first stage and households containing smokers were selected in the second stage. Two rounds of household data collection were implemented to obtain a representative sample of cigarette smokers in the 12 provinces. The target population included all non-institutionalized individuals aged 18 years old and above who resided in the 12 selected provinces and smoked manufactured cigarettes at least once per week. A list of census enumeration areas (EAs) and their respective population sizes in the selected provinces was obtained from the General Statistics Office. There were 7101 EAs in the 12 provinces, of which 694 contained fewer than 90 households. Because the ultimate goal of the survey was to reach 25 cigarette smokers in each EA, and the proportion of households with at least one regular smoker was estimated at 27% in 2006 (authors’ calculations based on Vietnam Household Living Standard Survey 2006), we excluded from the sampling frame any EAs with fewer than 90 households. Thus, in the first sampling stage, we randomly selected 125 EAs out of 6407 EAs with at least 90 households using a systematic probability proportional to size (PPS-WOR) sampling method without replacement. 

During the first round of household data collection, all households in the selected 125 EAs were canvassed to determine those with one or more members who smoked manufactured cigarettes at least once per week. Statistics staff at the district level conducted this listing phase in each EA from August to September 2011, and the research team subsequently randomly re-sampled 10% of the list as a quality check from September to November 2011. It was important to create an accurate list of all households with smokers in the selected 125 EAs in order to draw a representative sample of all smokers in the next stage.

In the second stage of sampling, we randomly selected 25 to 35 smokers from each EA depending on the number of smoking households residing in that EA. For EAs with fewer than 25 smokers, we included all of them in the sample. The final sample includes 3125 smokers. The face-to-face interviews were conducted by staff members from district statistics offices (who were trained and supervised by the research team) and by several researchers from Hanoi University of Commerce. During the interviews, the interviewers also asked for the participants’ permission to retain the last packs they purchased. The second round of data collection began in March 2012 and ended in June 2012. 91% of the selected smokers in round two of the household survey completed a survey questionnaire, yielding 2852 eligible responses and 2798 cigarette packs. Sampling weights were computed as the inverse of the product of the sampling probabilities for the selection of EAs and smokers.

#### 3.1.3. Ethics

Written informed consent was obtained from all survey respondents. The research protocol and questionnaire were approved by the Institutional Review Boards at the National Institute of Occupational and Environmental Health in Vietnam and at Duke University. The identifying information was not collected at the time of the interview; therefore, all data were anonymous. The identifying household location was subsequently excluded from the database before data analysis, so that subject anonymity and confidentiality were maintained.

### 3.2. Statistical Analysis

Descriptive analysis was conducted using Stata 14. Given that the observations were drawn to obtain a representative sample from the 12 surveyed provinces, the sample weights were used in all analyses. Since this is a cross-sectional survey analysis, the last pack purchased was assumed to be the regular brand consumed by respondents. Therefore, characteristics of the last pack, such as packaging, tax stamp, origin country, price, and sources of the pack, were used to infer characteristics of cigarettes that the respondents consumed throughout the year. This is a standard approach in survey analysis to account for any other random deviation from the smokers’ usual behavior. We chose this approach instead of asking about the packs that respondents purchased in the past year because self-reported data are inherently prone to recall bias, and we believe that respondents have incentives to underreport their consumption of illicit products. 

### 3.3. Estimation of Government Revenue Loss

To obtain our estimate of illicit cigarettes consumed at the national level, we scale the estimated number of illicit cigarettes consumed in the 12 provinces surveyed by the market share of those provinces in the national market, assuming that the share of illicit cigarettes in the total (legal and illegal) cigarette market of the surveyed provinces is the same as that in the rest of the country.

(1) $$I_{n}=\frac{I_{p}}{M_{p}}$$

where $I_{n}$ is the volume of illicit cigarettes in the country, $I_{p}$ is the volume of illicit cigarettes consumed in the 12 surveyed provinces, and $M_{p}$ is the share of the national total that is consumed in those provinces. $M_{p}$ was computed from GATS Vietnam 2010. 

GATS Vietnam 2010 is a nationally representative survey conducted by the Vietnam General Statistics Office as a component of the Global Tobacco Surveillance System. The details of GATS 2010 have been described elsewhere [13]. Since GATS was not designed to collect information about illicit cigarettes, its questionnaire allowed us to track only two illicit cigarette brands among multiple illicit brands consumed by Vietnamese smokers and did not include pack examination. It did, however, enable us to scale up our estimates from the VITA 2012 to the national level. The assumption is that the share of illicit cigarette consumption in our sample was similar to that of the country. Estimates of the prevalence of the two illicit brands from GATS 2010 serve as a check for this assumption.

The tax revenue lost due to illicit cigarette consumption is estimated as

(2) $$Revenue\;Loss=I_{n}\times P_{n}\times T$$

where $P_{n}$ is the average price of a pack of illicit cigarettes estimated from the VITA 2012, and T is the effective tax rate on domestically manufactured cigarettes. Based on the tax structure in 2012 (Table A1) and according to our back-of-the envelope calculation, the effective tax rate would be 41%, 39%, and 37% if the retail margin were 10%, 15%, and 20%, respectively. The exchange rate of July 2012 (1 VND = 0.00005 USD) was applied to convert the cigarette price from VND to USD [14]. 

## 4. Findings

In this section, we first characterize illicit cigarette consumption by region, origin country, distribution channel, and price. We then report the estimates of tax revenue lost to the illicit cigarette trade and discuss our findings in relation to the tobacco industry’s estimates.

### 4.1. Characterization of Illicit Cigarette Consumption

#### 4.1.1. Illicit Cigarette Consumption by Geographic Region

Table 1 provides a picture of total cigarette consumption in Vietnam. Panel A shows the shares of legal and illegal cigarettes. Panel B focuses on the distribution of illegal cigarettes by geographic region. 

Estimates from the VITA 2012 data indicated that 20.7 of packs purchased in the 12 surveyed provinces were illegal (Table 1, Panel A, Column 1). The majority of smokers of illicit cigarettes lived in the South. Although accounting for only 37% of the country’s population [15], the South consumed approximately 87% of the total illicit cigarettes nationwide. The North and the Central regions together accounted for only around 13.0% of the black market (Table 1, Panel B, Column 1). 

#### 4.1.2. Market Share and Origin Countries of Illicit Brands

By obtaining and investigating the most recently consumed packs, VITA 2012 uniquely provided credible information on points of sale and market shares of each illicit brand (Table 2). We documented 12 illicit brands circulating on the market. Columns (1) and (2) reported their shares in the total market, which includes both licit and illicit cigarettes, and the illicit market, respectively.

The most popular smuggled brands were Jet and Hero, owned by the Indonesian tobacco company Sumatra Tobacco Trading Company, which did not have a joint venture with the VTA [16,17]. These two brands made up 52% and 33% of the illicit market, respectively, and accounted for roughly 10% and 6% of the total consumption. In the total cigarette market, they stood as the brands with the third and fifth largest shares. The next top three brands, Esse from South Korea, 555 from the United Kingdom, and Nelson from an unidentified source, accounted for much smaller market shares. The top five made up roughly 98% of the illicit market and 19% of the general market. Other brands, including well-recognized international brands such as Marlboro and Mild Seven, had only minor shares.

#### 4.1.3. Distribution Channels

Convenience stores apparently served as the main point-of-sale outlets for smokers of illicit tobacco (72.2%). The second and third most important channels were through tea stalls/street vendors and coffee houses, respectively. These three points of sale accounted for approximately 93% of total illicit cigarette sales. Small percentages of smokers bought illegal cigarettes from cigarette stores (3.6%), flea markets (2.2%), or other places (0.1%). Our results indicate that no smoker reported buying tax-avoided or tax-evaded cigarettes from duty-free shops, supermarkets, or abroad (Table 3). 

#### 4.1.4. Prices of Illegal Cigarettes in Comparison with Legal Cigarettes

The average price of a pack of legal cigarettes cost 50 cents (USD) on average, while illegal packs cost 80 cents on average. Illicit cigarettes were 51% more expensive than licit cigarettes (Table 4, Panel A). Smokers in the North and the Central regions were willing to pay as much as 2.27 and 1.75 times the price of legal products in order to purchase illegal ones (Table 4, Panels B and C). The discrepancy was smallest in the South (1.28 times), but is still relatively large (Table 4, Panel D).

Prices of illegal products were significantly and consistently higher than prices of legal ones in all regions. This phenomenon led us to compare the prices of illegal and legal cigarettes of the same brands (Table 4, Panels E and F). 555 and Marlboro were the only two brands for which both legal and illegal versions were consumed by the VITA respondents. The legal 555 and Marlboro were domestically manufactured by a joint venture between British American Tobacco and Philip Morris International with the Vietnam National Tobacco Corporation (VINATABA), a state-owned corporation. The smuggled 555 and Marlboro accounted for 4.5% and 0.88% of the illicit market, respectively (see Table 2). Their prices were 34% and 48% more expensive than the Vietnamese-made Marlboro and 555 cigarettes, respectively. 

**Figure 1:**
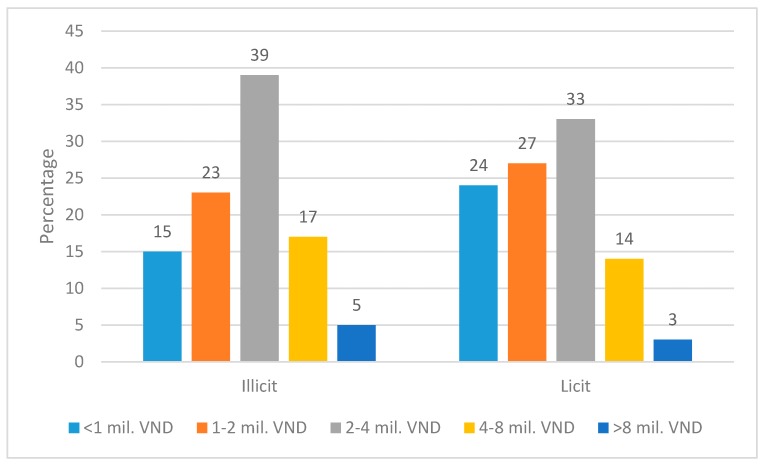
Monthly Income Distribution of Illicit/Licit Cigarette Smokers.

Those who consumed illicit cigarettes reported higher income levels that those who consumed legal cigarettes: 22% of illicit cigarette smokers belonged to the two highest income groups while this share was 17% among licit cigarette smokers; and only 38% of illicit cigarette smokers had income lower than 2 million VND/month (equivalent to $100/month) while this share was 51% among licit cigarette smokers (Figure 1).

### 4.2. Estimated Government Revenue Loss Caused by the Illicit Cigarette Trade

We estimate that between 724 and 731 million illicit cigarette packs circulated in the market in 2012 (Table 5). We estimate the tax revenue loss due to cigarette smuggling to be between $223 and $295 million USD given that retail margins range from 10% to 20% of the prior-sale price. 

As shown in Column (2) of Table 1, the analogous estimates from GATS 2010 are strikingly close to the estimates from VITA 2012. This similarity validates the assumption that the share of illicit cigarettes in the surveyed provinces was similar to the rest of the country, which supports the credibility of the revenue loss caused by illicit trade estimated in this section.

## 5. Discussion

This study uses a primary dataset from VITA 2012 to examine the magnitude and characteristics of illicit cigarettes in Vietnam. The results indicate that 20.7% of packs purchased were illegal, and between 724 and 731 million illicit cigarette packs circulated in the Vietnamese market in 2012. The most popular illicit brands were Jet and Hero, which made up almost 90% of the illicit market. According to the Vietnam Ministry of Industry and Trade, these two brands originated in Indonesia, were legally imported by Cambodia, and were then smuggled into Vietnam [11]. Singapore has been known as a transit point for other smuggled cigarettes in the region [18]. Convenience stores were the main channel through which the illicit products reached smokers, followed by street vendors and coffee shops. The evidence also indicates that illicit cigarettes were 51% more expensive than legal cigarettes.

Our estimate of illicit cigarette consumption is approximately 20% lower than the industry’s estimate of the illicit trade, which was 900 million packs, in the same year. The results suggest that this illicit trade prevented the collection of between $223 and $295 million USD in government revenue in 2012. These estimates of government revenue loss are 10% to 30% lower than the estimated loss calculated by the VTA [11], which was used for tax policy development in Vietnam. These findings support the observation that the tobacco industry tends to exaggerate the illicit trade problem in order to advocate against public policies such as tax increases and graphic health warnings. 

It is important to note that among the three main sources of illicit cigarettes (convenience/grocery stores, tea stalls/street vendors, and coffee houses), convenience/grocery stores and coffee houses have fixed locations and are legal economic enterprises. The government reviews their activities and grants them sale permits every year. Tea stalls and street vendors are mobile and thus more difficult to monitor [19]. Unlike the situation that has been documented for many other countries, no survey respondents bought illegal cigarettes from duty-free shops or from abroad. Smuggled cigarettes were probably so easy to find that smokers did not need to count on other sophisticated methods to obtain them. This finding suggests an important policy implication for Vietnamese law enforcement: inspection of cigarette retailers, especially convenience stores and coffee shops, is likely to be a cost-effective measure to detect illicitly traded cigarettes. The fact that three out of every twenty packs purchased from convenience stores were sold illegally, so this suggests a particular target for law enforcement efforts.

Last but not least, our results suggest that Vietnamese smokers do not choose to smoke illicit cigarettes because of lower cost. Quite the opposite, our survey indicates that illicit cigarettes are higher priced than licit cigarettes in every region and even within the same brand, and illicit cigarettes are more likely to be consumed by better-off smokers. This stands in contrasts to the Vietnamese tobacco industry’s claim that the illicit trade was caused by the high price of legal cigarettes [11]. 

One potential explanation is that the driver of illicit cigarette trade in Vietnam is determined by both demand and supply factors. From the demand side, Vietnamese consumers usually perceive that foreign-made products are of superior quality to the locally made products [20]. The same perception applies to cigarettes. Smuggled cigarettes are considered better in quality, safer, or “cooler” by smokers and therefore can command higher prices. The 555-brand cigarettes that were smuggled into the country were 75% more expensive and considered to be of higher quality than the 555 that were produced in Vietnam [21]. A parallel case may be found in Iran [22]; Heydari et al. (2010) has noted that illegal cigarettes in Tehran were higher in price because smokers believed that the illegal products were of higher quality. However, whether this perception is accurate is open to question. Of the illicit cigarette packs collected in the survey, 88.5% did not display information about their ingredients, the location, or country of manufacture. Thus, it is impossible to know whether they follow any quality control procedure and safety standard. Besides, none of the illicit packs carried any health warnings in Vietnamese, which prevented consumers from being aware of the toxic contents and health risks of smoking. The inherent perception of foreign-made products’ superior quality is enhanced by transnational tobacco companies. From the supply side, transnational tobacco companies carefully developed a price structure to ensure that the smuggled cigarettes were perceived as top-quality products. That pricing strategy lies among a set of marketing and business strategies employed by the industry to maximize their total profits from both licit and illicit markets [5,18]. 

Using the tax structure current in 2012, a back-of-the-envelope calculation suggests that the excise tax rate could have been raised from the 2012 rate of 65% of the factory price to 85% without causing the average price of legal cigarettes to exceed that of illicit cigarettes; and therefore, the excise tax increases from 65% to 70% of the factory price in 2016 which should not make an average legal cigarette pack more costly than an average illicit cigarette pack.

The study was subject to limitations. First, the estimation of illicit cigarettes at the national level is conducted based on the assumption that the distribution of illicit cigarette consumption is similar countrywide as in the 12 surveyed provinces. However, the comparison of the shares of illicit cigarette consumption estimated from VITA and GATS data supports this assumption. Second, the information about the cigarette packs at the last purchase is assumed to reflect characteristics of usual packs consumed by the respondents. Although this assumption is standard, the packs at the last purchase might not be exactly the same as the packs consumed throughout the year by the respondents. However, we expect departures from the last pack sampled and the cigarette brand regularly chosen by the smoker to be random across all respondents. Thirdly, the policy implications of these results regarding the effect of raising tobacco taxes on illicit cigarette consumption are constrained by the fact that only cross-sectional data are available. It would be useful if another later survey with the same design were conducted and combined with VITA to create a panel data to provide estimates of the effects of recent tobacco-related policies in Vietnam, such as raising taxes and implementing graphic health warnings, on illicit cigarette consumption. In spite of the limitations noted, the study makes an important contribution to the literature and to policy making by providing a comprehensive characterization of the illicit cigarette market and an independent estimate of illicit cigarette consumption in Vietnam. 

In summary, the results of our survey cast doubt on previous estimates of illicit cigarette consumption, as well as on the resulting estimates of government losses, that have been offered by the Vietnam Tobacco Association and used for policy development by the government. Our study also indicates that Vietnamese smokers do not choose to smoke illicit cigarettes because of lower prices given that they were willing to pay much higher prices for illicit products. In addition, the fact that most illegal cigarettes were purchased from neighborhood convenience stores suggests that weak law enforcement could be the main reason for the high market share of illicit cigarettes in Vietnam. 

## Figures and Tables

**Table 1:** Distribution of illicit cigarettes in Vietnam market (%).

	VITA 2012	GATS 2010
	(1)	(2)
**Panel A: Distribution of Cigarette Consumption by Legal Status**		
Legal	79.3	79.8
Illegal	20.7	20.2
**Panel B: Distribution of Illicit Cigarette Consumption by Region**		
North	7.1	2.4
Central	5.6	5.6
South	87.3	92.0

Notes: Estimates from VITA 2012 and GATS 2010.

**Table 2:** Market share and country source of illegal cigarettes.

Brand	Share in the Total Cigarette Market	Share in the Illicit Cigarette Market	Country Source
(1)	(2)	(3)	(4)
Jet	10.1	52.1	from Indonesia through Cambodia to Vietnam
Hero	6.4	32.8	from Indonesia through Cambodia to Vietnam
Esse	0.99	5.1	South Korea
555	0.88	4.5	UK brand
Nelson	0.61	3.1	unidentified
Mey	0.17	0.88	Vietnam
Marlboro	0.09	0.49	unidentified
Elephant	0.07	0.37	unidentified
Mild Seven	0.05	0.25	Japan
Gudang Garam	0.04	0.20	Indonesia
Zest	0.02	0.11	South Korea
Sundays	0.02	0.09	unidentified

Notes: Estimates from VITA 2012.

**Table 3:** Sources of cigarettes in the last purchase (%).

Sources of Cigarettes	VITA 2012
Cigarette store	3.6
Convenient/grocery store	72.2
Duty free shop	0.00
Tea stall/street vendor	17.2
Flea market	2.2
Restaurant/Eating place	0.10
Coffee house	5.5
Supermarket	0.00
Buy from abroad	0.00
Other	0.11

Notes: Estimates from VITA 2012.

**Table 4:** Prices of illicit and licit cigarette packs ($US).

	Mean	95% Confidence Interval		Price Ratio
		Lower Bound	Upper Bound	
	(1)	(2)	(3)	(4)
**Panel A: Overall Country**				
Illegal	0.78	0.75	0.82	1.51
Legal	0.52	0.49	0.55	
**Panel B: North**				
Illegal	1.18	0.93	1.43	2.27
Legal	0.52	0.48	0.55	
**Panel C: Central**				
Illegal	0.77	0.61	0.94	1.75
Legal	0.44	0.40	0.49	
**Panel D: South**				
Illegal	0.77	0.74	0.79	1.28
Legal	0.60	0.54	0.67	
**Panel E: Legal 555 vs. illegal 555**				
Illegal	1.58	1.81	1.36	1.34
Legal	1.18	1.23	1.10	
**Panel F: Legal Marlboro vs. illegal Marlboro**				
Illegal	1.51	1.99	1.04	1.48
Legal	1.02	1.05	1.00	

Notes: Estimates from VITA 2012.

**Table 5:** Estimated Tax Revenue Loss Caused by Illicit Cigarette Trade in 2012.

	Mean	95% Confidence Interval	
		Lower Bound	Upper Bound
	(1)	(2)	(3)
**Panel A. Estimated Number of Illicit Packs Consumed (Million)**			
Illicit packs	728	724	731
**Panel B. Estimated Tax Revenue Loss (US$)**			
10% retail margin	271,050,000	294,550,000	247,750,000
15% retail margin	257,800,000	280,200,000	235,650,000
20% retail margin	244,600,000	265,800,000	223,600,000

Notes: Estimates from VITA 2012.

## Appendix A

**Table A1:** Tobacco Tax Rates, 1990–2019.

Year	Special Consumption Tax (%)				Value-Added Tax (%)	Compulsory Contribution to Tobacco Control Fund (%)
	Cigarettes			Cigars		
	Filter, Mainly Made With		Non-Filter			
	Imported material	Domestic Material				
(1)	(2)	(3)	(4)	(5)	(6)	(7)
1990–1993	50.0	50.0	40.0	40.0	0.0	
1993–1995	70.0	52.0	32.0	32.0	0.0	
1996–1998	70.0	52.0	32.0	70.0	0.0	
1999–2005	65.0	45.0	25.0	65.0	10.0	
2006–2007	55.0	55.0	55.0	55.0	10.0	
2008–2013	65.0	65.0	65.0	65.0	10.0	
2013–2016	65.0	65.0	65.0	65.0	10.0	1.0
2016–2019	70.0	70.0	70.0	70.0	10.0	1.5

Notes: Sources: Guindon et al. (2010) and SEATCA (2017) [23].
